# Cardiac Contractility Modulation and Arrhythmic Burden in Heart Failure: Mechanistic Rationale, Clinical Evidence, and Future Perspectives

**DOI:** 10.3390/jcdd13080362

**Published:** 2026-08-01

**Authors:** Andrea Palermi, Silvio Saraullo, Massimiliano Faustino, Daniele Sacchetta, Roberta Magnano, Lorenzo Mazzocchetti, Stefano Guarracini, Massimo Di Marco, Nanda Furia, Sabina Gallina, Giulia Renda

**Affiliations:** 1 Department of Neuroscience, Imaging and Clinical Sciences, G. d’Annunzio University of Chieti-Pescara, Via dei Vestini 31, 66100 Chieti, Italy; apalermi96@gmail.com (A.P.); silvio.saraullo.ss@gmail.com (S.S.); sabina.gallina@unich.it (S.G.); 2 Arrhythmology Unit, “SS. Annunziata” Hospital, Via dei Vestini 31, 66100 Chieti, Italy; m.faustino@asl2abruzzo.it; 3 Department of Cardiology, “S. Pio da Pietrelcina” Hospital, 66054 Vasto, Italy; nanda.furia@asl2abruzzo.it; 4 Department of Cardiology, “Santo Spirito” Hospital, 65124 Pescara, Italy; danielesacchetta@gmail.com (D.S.); roberta.magnano@asl.pe.it (R.M.); massimo.dimarco@asl.pe.it (M.D.M.); 5 Department of Cardiology, “Pierangeli” Hospital, 65124 Pescara, Italy; lorenzomazzocchetti90@gmail.com (L.M.); stefanoguarracini@yahoo.it (S.G.); 6 University Cardiology Unit, Heart Department, “SS. Annunziata” Hospital, Via dei Vestini 31, 66100 Chieti, Italy

**Keywords:** cardiac contractility modulation, heart failure, arrhythmic burden, ventricular arrhythmias, atrial fibrillation, implantable cardioverter-defibrillator, cardiac remodeling

## Abstract

Cardiac contractility modulation (CCM) is an implantable device-based therapy that delivers biphasic, non-excitatory electrical signals to the ventricular myocardium during the absolute refractory period. By enhancing contractile performance without inducing depolarization or altering ventricular activation, CCM acts as bioelectronic myocardial conditioning. Current evidence supports its use in selected patients with symptomatic heart failure, reduced or mildly reduced left ventricular ejection fraction, narrow QRS duration, persistent symptoms despite guideline-directed medical therapy, and no indication for cardiac resynchronization therapy. In this population, CCM improves functional status and quality of life, whereas evidence for reductions in mortality or recurrent heart failure hospitalization remains less definitive. Whether CCM also reduces arrhythmic burden remains uncertain. Candidates for CCM frequently exhibit atrial and ventricular remodeling, neurohormonal activation, implantable cardioverter-defibrillators, and vulnerability to atrial fibrillation, ventricular arrhythmias, and device therapies. Mechanistically, CCM may render the failing myocardium less arrhythmogenic through coordinated effects on calcium handling, electromechanical remodeling, fibrosis-related substrate, contractile efficiency, and heart-failure stability. However, pivotal trials were not designed to assess arrhythmic endpoints, leaving the relationship between CCM and arrhythmic burden insufficiently characterized. This review summarizes CCM evidence, mechanistic rationale, available arrhythmic signals, device-related considerations, and future research priorities for prospective studies in this evolving field.

## 1. Introduction

Heart failure and arrhythmias are tightly linked clinical phenotypes. In the failing heart, atrial and ventricular remodeling create a proarrhythmic substrate through the convergence of structural, electrical, and systemic abnormalities. Chamber dilation, wall stress, and fibrosis promote conduction heterogeneity and re-entry, whereas impaired calcium cycling, neurohormonal activation, inflammation, renal dysfunction, and electrolyte instability increase vulnerability to triggered activity and rhythm instability. These abnormalities increase susceptibility to ventricular arrhythmias through partially distinct mechanisms. Fibrosis, scar heterogeneity, chamber dilation, and regional conduction slowing favor organized re-entrant circuits that may sustain monomorphic ventricular tachycardia (VT), whereas impaired calcium handling, repolarization dispersion, ischemia, adrenergic activation, and acute metabolic or electrolyte disturbances may promote triggered activity, polymorphic ventricular tachycardia, or degeneration into ventricular fibrillation (VF). In parallel, atrial stretch, elevated filling pressures, autonomic imbalance, inflammation, and atrial fibrosis promote atrial electrical and structural remodeling, thereby facilitating atrial fibrillation (AF). Once established, arrhythmias may worsen hemodynamics, symptoms, quality of life, hospitalization risk, the burden of implantable cardioverter-defibrillator (ICD) therapies, and survival, while also carrying substantial socioeconomic and public-health implications related to recurrent hospital admissions, device therapies, and long-term healthcare resource use [1,2].

Current strategies to reduce arrhythmic risk in heart failure act at different levels: guideline-directed medical therapy (GDMT) improves the disease substrate, antiarrhythmic drugs and catheter ablation target specific triggers or arrhythmogenic substrates, and ICDs prevent sudden cardiac death by terminating malignant ventricular arrhythmias. In this context, cardiac contractility modulation (CCM) occupies a distinct conceptual space, as it may influence arrhythmic vulnerability through myocardial substrate modification rather than direct rhythm suppression or arrhythmia termination. CCM delivers non-excitatory electrical signals during the absolute refractory period and enhances the strength of cardiac contraction by improving calcium handling.

This enables a reduction in symptoms and an increase in functional capacity, exercise tolerance, and health-related quality of life in selected patients with symptomatic heart failure (HF), reduced or mildly reduced ejection fraction, narrow QRS duration, and persistent limitation despite GDMT [3,4,5,6,7,8]. However, CCM was not developed as an antiarrhythmic therapy, and pivotal studies were not designed to quantify the burden of AF or PVC, non-sustained ventricular tachycardia (NSVT), sustained VT/VF, antitachycardia pacing (ATP), or ICD shocks as primary endpoints [5,6,7,8].

The key question is whether CCM-related substrate modification translates into measurable reductions in atrial and ventricular arrhythmic burden, beyond its established effects on heart-failure symptoms and functional status.

To address this question, we performed a structured narrative literature search in PubMed/MEDLINE and Scopus, supplemented by reference screening of key trials, registries, reviews, mechanistic studies, and case reports. We aimed to include all identified studies specifically reporting arrhythmic-burden changes after CCM, including ventricular and supraventricular arrhythmias.

This review summarizes CCM positioning, mechanistic rationale, available supraventricular and ventricular arrhythmic signals, device-related monitoring issues, and methodological priorities for future studies.

## 2. Current Role of CCM in Heart Failure

The clinical evidence supporting cardiac contractility modulation (CCM) derives mainly from the FIX-HF program, pooled analyses, and real-world registries (Table 1). Overall, these studies define CCM as a therapy primarily aimed at improving symptoms, exercise capacity, functional status, and health-related quality of life in selected patients with symptomatic heart failure (HF), narrow QRS duration, and reduced or mildly reduced left ventricular ejection fraction (LVEF), rather than as an intervention with established antiarrhythmic efficacy [4,5,6,7,8,9,10,11].

The randomized FIX-HF program progressively shaped the current evidence base. In FIX-HF-4, early double-blind crossover data showed improvements in peak oxygen consumption and HF-related quality of life among patients with symptomatic heart failure with reduced ejection fraction (HFrEF) and narrow QRS duration [5]. FIX-HF-5 subsequently confirmed the safety of CCM, although it failed to meet its prespecified primary efficacy endpoint based on ventilatory anaerobic threshold in the overall population [6]. However, favorable secondary outcomes suggested that patients with moderately reduced LVEF might derive greater benefit. This hypothesis was prospectively tested in FIX-HF-5C, in which CCM improved exercise capacity, quality of life, NYHA functional class, and 6 min walk distance in patients with LVEF 25–45% and QRS duration < 130 ms [7].

A favorable short-term signal for cardiovascular death or HF hospitalization was observed in FIX-HF-5C, while CCM-REG and other observational cohorts reported lower HF hospitalization rates after implantation [7,10,11,12,13]. This may reflect improved contractile efficiency, forward flow, and reverse remodeling, with lower filling pressures and reduced susceptibility to recurrent congestion. However, this mechanism remains inferential, and a causal reduction in HF hospitalization has not been conclusively demonstrated, because randomized evidence is underpowered and most long-term data derive from uncontrolled pre–post comparisons. Moreover, randomized evidence has not established a reduction in all-cause mortality [9].

This clinical profile has been reinforced by pooled and observational data. An individual-patient-data meta-analysis confirmed consistent improvements in peak VO_2_, 6 min walk distance, and Minnesota Living with Heart Failure Questionnaire (MLWHFQ) score across randomized CCM trials [8]. Similarly, CCM-REG showed sustained improvements in NYHA class, quality of life, LVEF, and HF/cardiovascular hospitalization rates, with observed survival exceeding that predicted by risk models such as the Meta-Analysis Global Group in Chronic Heart Failure (MAGGIC) score in selected LVEF strata [10,11]. Additional long-term clinical experience and contemporary updates have further supported this symptom- and function-improving profile while emphasizing persistent uncertainty regarding hard clinical and arrhythmic outcomes [12,13]. However, these registry findings remain observational and model-based, and are therefore vulnerable to residual confounding, selection bias, and pre–post comparison bias.

Furthermore, small observational studies suggest that CCM may improve myocardial deformation and mechanical efficiency beyond conventional changes in LVEF. Masarone et al. reported improvements in global longitudinal strain (GLS) and echocardiographically estimated myocardial mechano-energetic efficiency, mainly driven by increased stroke volume without a concomitant rise in heart rate [14]. Pressure–strain loop analyses provided complementary evidence of higher global work index, constructive work, and work efficiency, with lower wasted work, suggesting more effective ventricular mechanical performance, even when GLS remained unchanged [15,16]. Subsequent data also confirmed favorable changes in GLS during active CCM therapy [17].

Subgroup analyses suggest that CCM may benefit both HFrEF and lower-range HFmrEF, with potentially greater functional improvement at LVEF 35–45%, possibly reflecting less advanced remodeling and greater contractile reserve, although this remains unproven [7,10]. Sex-specific evidence is limited by the underrepresentation of women; however, an individual-patient-data meta-analysis found no significant sex–treatment interactions for peak VO_2_, quality of life, or 6 min walk distance [8]. A small observational study suggested greater GLS improvement in women, but this finding remains exploratory [17]. Similarly, no significant age–treatment interaction has been demonstrated, with functional improvements observed in patients both ≤60 and >60 years [8]. Therefore, neither sex nor chronological age should independently determine CCM eligibility; instead, frailty, comorbidity burden, procedural risk, life expectancy, and the likelihood of meaningful symptomatic benefit should inform patient selection, although their relative predictive value requires prospective validation.

Guideline and regulatory positioning consequently remain cautious. CCM should still be viewed primarily as a symptom- and function-improving therapy in selected patients with symptomatic HF, narrow QRS duration, and reduced or mildly reduced LVEF [18,19]. Emerging data suggest that CCM may also have relevance in higher-LVEF phenotypes. In the CCM-HFpEF pilot study, Linde et al. reported improved health status in symptomatic heart failure with preserved ejection fraction (HFpEF), supporting the hypothesis that non-excitatory myocardial conditioning may extend beyond traditional HFrEF/HFmrEF populations [20]. However, this evidence remains preliminary, and larger prospective studies, including AIM HIGHer, are needed to determine whether CCM improves symptoms, functional capacity, health status, and clinical outcomes in patients with higher ejection fraction [21].

Although CCM is primarily positioned for symptomatic HF patients with narrow QRS duration and no indication for cardiac resynchronization therapy (CRT), adjunctive use in selected patients already treated with CRT, particularly CRT non-responders, has also been explored. This remains supported by limited evidence and should be regarded as an individualized strategy rather than an established indication [22]. Importantly, CCM should not be considered a substitute for implantable cardioverter-defibrillator (ICD) therapy or CRT, and current evidence does not support definitive claims of mortality reduction, recurrent hospitalization reduction, or arrhythmic-burden reduction [18,19].

This distinction is particularly relevant because many CCM candidates already have ICDs and a clinically meaningful atrial or ventricular arrhythmic burden. Therefore, any reduction in atrial fibrillation (AF), premature ventricular complexes (PVCs), non-sustained ventricular tachycardia (NSVT), sustained ventricular tachycardia/ventricular fibrillation (VT/VF), antitachycardia pacing (ATP), or ICD shocks after CCM should be interpreted cautiously and should not be equated with established antiarrhythmic efficacy.

**Table 1:** Established clinical evidence for CCM in HF and its relevance to the arrhythmia issue.

Study	HF Evidence	Arrhythmic Assessment	Relevance to the Arrhythmia Issue
FIX-HF-3 experience [4]	Early feasibility and safety experience in advanced symptomatic HF with normal/narrow QRS.	Holter and safety-oriented assessment; no predefined arrhythmic efficacy endpoint.	Supports feasibility and absence of a major proarrhythmic signal, not burden reduction.
FIX-HF-4 [5]	Randomized double-blind crossover trial showing improved peak VO_2_ and HF-related quality of life.	No predefined arrhythmic efficacy endpoint.	Supports functional benefit; does not address arrhythmic burden.
FIX-HF-5 [6]	Larger randomized trial; safety endpoint met; primary VAT endpoint neutral overall; favorable secondary functional signals.	Holter and device-safety data; no systematic PVC/NSVT/VT burden assessment.	Supports electrical safety, not antiarrhythmic efficacy.
FIX-HF-5C [7]	Confirmatory RCT in NYHA III/ambulatory IV, LVEF 25–45%, QRS < 130 ms; improved exercise capacity, QoL, NYHA class, and 6MWD; favorable short-term HF outcome signal.	Not powered for arrhythmic endpoints.	Relevant target population often overlaps with ICD candidates, but arrhythmic efficacy remains untested.
FIX-HF-5C2 [23]	Contemporary two-lead system supporting functional improvement and simpler device configuration.	Relevant to AF/unreliable atrial sensing and practical device delivery but not designed for rhythm efficacy.	Supports feasibility in broader rhythm/device contexts; no proof of burden reduction.
Giallauria et al. [8]	Confirmed improvements in peak VO2, 6MWD, and MLWHFQ across randomized CCM trials.	Arrhythmic outcomes are not the focus.	Supports HF-stabilization rationale, not direct arrhythmic efficacy.
Nadeem et al. [9]	RCT meta-analysis did not establish definitive all-cause mortality reduction.	No arrhythmic endpoint focus.	Reinforces that hard-outcome evidence, including arrhythmic death, remains insufficient.
Anker et al.; Kuschyk et al. [10,11]	Registry data showing sustained symptom/LVEF improvement and fewer HF/CV hospitalizations; survival better than predicted in selected strata.	Arrhythmic burden not systematically adjudicated.	Supports indirect HF-stabilization hypothesis; observational and confounded.
Yucel et al. [24]	Long-term single-center data suggesting durable functional and echocardiographic benefit across baseline LVEF subgroups.	No standardized arrhythmic endpoint.	Supports durability of HF response, but not arrhythmia-specific efficacy.
Linde et al.; AIM HIGHer [20,21]	Pilot and ongoing evidence in higher-LVEF phenotypes, including HFpEF.	No established rhythm-efficacy endpoint.	Relevant to future AF/HFpEF phenotyping; currently hypothesis-generating.
Röger et al. [25]	First clinical experience; 5 patients with HFrEF and permanent AF.	Feasibility of CCM delivery in permanent AF.	Supports technical feasibility of CCM in patients with permanent AF, but does not demonstrate AF-burden reduction or rhythm-control efficacy.
Tint et al. [26]	Pilot experience in HFrEF patients with AF and previous CRT; improved LVEF, NYHA class, and exercise tolerance at 1 year.	AF burden not quantified; no rhythm-efficacy endpoint.	Supports feasibility/clinical response in AF + prior CRT, not AF-burden reduction.
Shin et al. [27]	Single-patient report with improved HF status after CCM.	Longitudinal rhythm monitoring showed negligible AF burden after CCM.	Direct AF-burden signal, but very-low certainty and confounded by revascularization, GDMT, LVEF recovery, and antiarrhythmic exposure.
De Donno et al. [28]	Single-patient HFrEF report with symptomatic improvement after CCM.	Reduction in ventricular arrhythmic burden and no ICD therapies during follow-up.	Direct ventricular arrhythmia signal, but very-low certainty and not causal.

Abbreviations are listed in the Abbreviations section.

## 3. Mechanistic Pathways Linking CCM to Arrhythmia Modulation

The biological rationale linking CCM to arrhythmia modulation is grounded in its effects on the failing myocardial substrate. Across experimental, translational, and early clinical studies, CCM appears to influence several interconnected dimensions of the failing myocardial substrate (graphical abstract). These include calcium cycling and myocardial mechanics at the cellular level, molecular and structural remodeling, and broader effects on repolarization, autonomic balance, and HF stability [2,29,30,31,32,33,34,35,36,37,38,39]. These mechanisms do not prove antiarrhythmic efficacy, but they provide a coherent framework through which CCM could make the failing myocardium less vulnerable to atrial and ventricular arrhythmias. The following sections summarize the main mechanisms through which CCM could modulate arrhythmia susceptibility.

### 3.1. Reverse Electromechanical Remodeling

CCM has been consistently associated with reverse electromechanical remodeling of the failing myocardium. Experimental and translational studies suggest favorable modulation of calcium-handling pathways. In HF, calcium cycling is disrupted at multiple levels, including impaired sarcoplasmic-reticulum calcium reuptake, reduced SERCA2a activity, altered phospholamban regulation, ryanodine receptor dysfunction, and maladaptive sodium–calcium exchanger activity. The resulting cytosolic calcium instability and diastolic calcium leak favor delayed afterdepolarizations, PVCs, and ventricular tachyarrhythmias [30,33,34]. Chronic CCM has been shown to modulate calcium influx and improve the expression or regulation of key calcium-cycling proteins, including SERCA2a, phospholamban, ryanodine receptor-2, and the sodium–calcium exchanger [30,33,34]. Human myocardial biopsy studies similarly suggest partial normalization of calcium-handling and stretch-response gene expression after CCM exposure [31]. Importantly, these effects appear to occur without the β-adrenergic/cAMP-dependent increase in myocardial oxygen demand typical of conventional pharmacological inotropes [38,39]. CCM has also been associated with reverse molecular and structural remodeling. In preclinical models, CCM improved LV function, reduced chamber dilatation, attenuated extracellular-matrix remodeling, and partially reversed the maladaptive fetal gene program that characterizes the failing myocardial phenotype [30,31,32]. Human tissue studies have shown modulation of genes involved in sarcoplasmic-reticulum calcium handling, myocardial stretch response, cytoskeletal organization, and extracellular-matrix turnover [31]. Additional preclinical evidence suggests attenuation of interstitial and replacement fibrosis through reduced collagen production, fibroblast differentiation, and Transforming Growth Factor Beta 1 (TGF-β1)/Smad3 signaling [35]. More recent transcriptomic data in ischemic HFrEF suggest that CCM response is accompanied by a shift toward improved mitochondrial quality, proteostasis, cardiomyocyte structural integrity, together with attenuation of inflammatory pathways [36]. These findings suggest improved mitochondrial quality control, proteostasis, contractile architecture, and attenuation of maladaptive extracellular-matrix remodeling, although whether this signature is directly induced by CCM or reflects favorable reverse remodeling in responders remains uncertain.

The electrophysiological component of this remodeling is particularly relevant to arrhythmia modulation. In chronic HF models, CCM shortened pathologically prolonged QTc and ventricular effective refractory period, reduced VT inducibility, attenuated fibrosis, and partially restored potassium-channel and gap-junction proteins, including Kv4.3, KCNQ1, KCNH2, and connexin-43 [35]. These findings suggest that CCM may influence not only calcium-dependent triggered activity, but also the conduction and repolarization substrate required for re-entrant arrhythmias. In the failing ventricle, fibrosis, scar heterogeneity, myocyte disarray, reduced sodium-current availability, and gap-junction remodeling slow impulse propagation and increase spatial dispersion of conduction [40]. Connexin-43, the predominant ventricular gap-junction protein, is essential for coordinated cell-to-cell electrical coupling [41]; its reduced expression, lateralized distribution, altered phosphorylation, or heterogeneous organization may impair intercellular current flow, slow conduction velocity, and promote conduction heterogeneity [42]. In parallel, potassium-channel remodeling may contribute to delayed repolarization, repolarization dispersion, and electrical instability. Thus, improved calcium cycling may reduce triggered activity, whereas attenuation of fibrosis, matrix disorganization, ion-channel remodeling, and impaired gap-junction coupling may limit conduction heterogeneity and re-entry vulnerability. From this perspective, CCM-related modulation of connexin-43 and conduction biology may represent a functional correlate of arrhythmogenic substrate modification rather than a simple consequence of improved contractility [43]. However, whether CCM can meaningfully modify scar-related conduction properties or reduce re-entrant ventricular arrhythmias in humans remains unproven.

### 3.2. Contractile Efficiency and Mechano-Electric Feedback

A central biological effect of CCM is the improvement in myocardial contractile performance. Consistently, transcriptomic analyses in ischemic HFrEF have shown that clinical response to CCM is associated with up-regulation of sarcomeric, Z-disk, and cardiomyocyte structural genes, supporting a potential link between CCM response, improved contractile architecture, and mechano-electric efficiency [36]. Unlike conventional positive inotropes, CCM delivers non-excitatory signals during the absolute refractory period and enhances contractility without triggering a new depolarization or relying primarily on beta-adrenergic/cAMP-dependent stimulation. Experimental and translational studies suggest that this effect reflects more efficient electromechanical coupling, supported by improved calcium cycling, myofilament function, and contractile-protein phosphorylation. As a result, CCM may enhance contractile performance without the energetic penalty typical of pharmacological inotropic stimulation [2,37,38,39].

This distinction is clinically relevant. Conventional inotropes may increase arrhythmic risk by promoting calcium overload, adrenergic stimulation, higher oxygen demand, and electrical instability. CCM, by contrast, may improve systolic performance in a more substrate-stabilizing manner. By reducing wall stress, mechanical dispersion, and filling pressures, it could also attenuate stretch-dependent electrical instability. This could attenuate mechano-electric feedback, triggered activity, and re-entry susceptibility. CCM may also influence myofilament function and titin-based stiffness. In HFrEF, CCM has been associated with increased phosphorylation of troponin I, myosin-binding protein C, and titin, which may improve myofilament calcium sensitivity, contractile efficiency, diastolic recoil, and passive stiffness [2]. Improved sarcomeric mechanics and titin compliance provide a plausible link between better contractile efficiency and lower arrhythmia susceptibility. Still, CCM-related inotropism should be interpreted as a potential substrate-modifying mechanism, not as proven antiarrhythmic efficacy.

### 3.3. Neurohormonal and Autonomic Modulation

A further potential pathway is the indirect modulation of arrhythmia burden through improved autonomic balance. HF is characterized by sympathetic activation, parasympathetic withdrawal, and impaired baroreflex function, all of which increase vulnerability to atrial and ventricular arrhythmias, particularly when accompanied by elevated filling pressures and ventricular stretch [44,45,46].

CCM may influence this pathway through hemodynamic and neuroautonomic mechanisms. By improving contractile efficiency, congestion, and functional status, it may reduce neurohormonal activation and adrenergic triggers of arrhythmogenesis. Mechanistic observations also suggest that CCM may decrease muscle sympathetic nerve activity over time and interact with cardiac vagal afferent pathways, potentially improving sympathovagal balance [1,2].

Improved autonomic balance could theoretically reduce adrenergically mediated PVCs, AF burden, VT/VF, and ICD therapies. Nevertheless, no clinical study has shown that CCM reduces arrhythmic burden through autonomic modulation; this pathway remains a plausible contributor to HF substrate stabilization rather than proven antiarrhythmic efficacy.

## 4. Clinical Signals for Arrhythmic-Burden Reduction

The mechanistic evidence reviewed above supports the concept that CCM may render the failing myocardium less arrhythmogenic through reverse electromechanical remodeling, including improved calcium handling, reduced fibrosis-related substrate, enhanced contractile efficiency, more favorable repolarization biology, autonomic stabilization, and improved HF status. However, whether these substrate changes translate into fewer clinical arrhythmias, device-detected VT/VF episodes, ATP, or ICD shocks remains uncertain. The available clinical evidence should therefore be interpreted as signals of possible arrhythmia modulation rather than definitive proof of rhythm efficacy.

### 4.1. Supraventricular Arrhythmias

Atrial arrhythmias are common in symptomatic HF patients considered for CCM, and AF has important implications for sensing, patient selection, and therapy delivery. Importantly, AF is not a contraindication to contemporary CCM therapy. Earlier systems relied on atrial sensing, limiting their use in persistent or permanent AF. Initial experience showed that CCM delivery could be enabled through atrioventricular pacing or CRT-based strategies, allowing atrial pacing signals to serve as a surrogate for atrial sensing. In the small series by Röger et al., this approach was feasible in selected patients with HFrEF and permanent AF, although the study addressed technical delivery rather than AF-burden reduction or rhythm-control efficacy [25]. Contemporary two-lead systems no longer require atrial sensing and therefore allow CCM delivery in patients with persistent or permanent AF [23].

Additional observational experience has explored CCM in complex patients with HFrEF, AF, and previous resynchronization therapy. Tint and Micu reported improvements in LVEF, NYHA class, and exercise tolerance at 1 year, without major device-related safety concerns, suggesting that CCM may be clinically feasible in selected patients with AF and prior CRT. Consistently, CCM-REG showed improvements in quality of life and LVEF, together with lower HF hospitalization rates, among patients with AF, supporting the clinical benefit of CCM in this population [11]. However, AF burden was not quantified before and after CCM, and AF recurrence, rhythm-control interventions, or device-detected atrial arrhythmias were not assessed as predefined endpoints. Therefore, these data support feasibility and clinical response in AF/CRT populations, but not AF-burden reduction [26].

Evidence that CCM directly reduces supraventricular arrhythmias remains extremely limited. The most direct AF-burden signal is the case report by Shin et al., describing a patient with ischemic cardiomyopathy, paroxysmal AF, persistent HF symptoms, and implantable loop recorder monitoring [27]. After CCM implantation, functional status improved, LVEF recovered, and no AF recurrence was detected over approximately 1.5 years. This observation is clinically interesting because continuous monitoring was used, but its evidentiary strength is very low: pre-implant AF burden was not quantified, amiodarone exposure and discontinuation may have influenced rhythm trajectory, and coronary revascularization, GDMT optimization, reverse LV remodeling, and improved HF status are major confounders.

Overall, available clinical evidence supports the feasibility and clinical benefit of CCM in patients with AF, but not its use as a rhythm-control therapy. No randomized or adequately powered prospective study has demonstrated reductions in AF incidence, AF recurrence, AF burden, atrial tachyarrhythmia burden, or the need for rhythm-control interventions after CCM.

### 4.2. Ventricular Arrhythmias and ICD Therapies

Clinical evidence for CCM-related reduction in ventricular arrhythmias and ICD therapies remains limited and should be distinguished from evidence of electrical safety. Early studies asked mainly whether biphasic, non-excitatory signals delivered during the absolute refractory period were proarrhythmic. In FIX-HF-3 and subsequent feasibility experience, Holter-ECG monitoring and device assessment did not show an increase in ventricular or supraventricular tachyarrhythmias during CCM therapy [1,4]. Regulatory safety data similarly reported no arrhythmias induced during CCM delivery [23]. These findings support a non-proarrhythmic safety profile, rather than reductions in PVC burden, NSVT, sustained VT/VF, ATP, or ICD shocks.

Long-term observational studies provide additional but indirect information. In the severe HF cohort reported by Schau et al., ventricular arrhythmias remained clinically relevant despite CCM: ICD shocks occurred in six patients, including five appropriate shocks, and two patients underwent ablation for recurrent ventricular arrhythmias [47]. In the single-center long-term experience by Kuschyk et al., almost all patients had an ICD or underwent concomitant ICD implantation, 12 patients received appropriate shocks for VT/VF, and a composite of all-cause mortality plus first ICD-documented VT/VF occurred in 13.1% at 1 year and 32.1% at 3 years [48]. These studies show that CCM-treated patients remain at arrhythmic risk, but they did not quantify pre/post-burden, standardize ICD programming, or adjudicate ventricular arrhythmias as efficacy endpoints.

The most direct ventricular arrhythmia signal remains the case report by De Donno et al., describing a patient with HFrEF, ICD implantation, and high ventricular arrhythmic burden before CCM [28]. After CCM, the authors reported symptomatic improvement, marked reduction in ventricular arrhythmic events, no sustained arrhythmias requiring ICD intervention, and no ICD therapies at 6 months; device interrogation showed no relevant CCM-ICD interaction [28]. The report is clinically provocative because the reduction was captured in a patient with prior high ventricular burden and ICD monitoring. However, a single case cannot separate a CCM-related effect from regression to the mean, improved HF status, medication changes, or other cointerventions.

### 4.3. Device-Related Considerations: Interaction and Arrhythmic-Burden Monitoring

Device interaction is a practical issue when CCM is used in patients with ventricular arrhythmic risk. Many candidates already carry an ICD or S-ICD, or may require defibrillator protection because of reduced LVEF, previous ventricular arrhythmias, or high-risk substrate. Coexistence of CCM and defibrillator therapy matters for procedural safety and for interpretation of arrhythmic outcomes. Combined CCM and S-ICD therapy can be feasible when crosstalk testing, sensing assessment, defibrillation testing, stored electrogram review, and individualized programming are performed [49,50]. In the long-term experience by Roger et al., no clinically relevant interference affected device function when appropriate testing was used [49]; isolated reports of S-ICD oversensing or misclassification after CCM implantation show that compatibility must be actively verified [50].

CCM signals may be detected by the defibrillator sensing channel, potentially causing oversensing, rhythm misclassification, inappropriate therapies, or inaccurate estimation of ventricular arrhythmic burden [49,50]. Careful interaction testing and individualized programming are therefore required to preserve reliable VT/VF detection, avoid inappropriate ICD/S-ICD therapies or unnecessary CCM inhibition, and ensure accurate arrhythmic-burden assessment [49,50]. These effects improve monitoring reliability and therapy safety, but they do not prove that CCM reduces true ventricular arrhythmia occurrence.

Integrated CCM–ICD platforms may change this landscape. The investigational OPTIMIZER Integra CCM-D system combines CCM and ICD therapy in a single device and is being evaluated in INTEGRA-D (NCT05855135), a prospective, single-arm, multicenter program designed to assess whether a combined CCM–ICD system can treat induced VF and spontaneous VT/VF while delivering CCM for HF [51]. Follow-up is intended to evaluate defibrillation performance, device-related complications, inappropriate shocks, and continued CCM delivery. A single platform may reduce the need for separate pulse generators, simplify management, decrease cumulative procedural and lead-related burden, and integrate HF modulation, VT/VF detection, ATP/shock therapy, and rhythm monitoring. It could also standardize sensing, synchronized therapy delivery, endpoint definition, and quantification of VT/VF episodes, ATP, appropriate and inappropriate shocks, and burden normalized to device-observation time. Thus, integrated CCM–ICD systems may become an important research platform for arrhythmic-burden assessment, but their current value is technological and methodological rather than evidence of rhythm suppression.

## 5. Evidence Gaps and Future Research Directions

Arrhythmic-burden reduction remains a major evidence gap because existing CCM studies were designed to assess symptoms, functional capacity, quality of life, and safety rather than rhythm efficacy. It remains unknown whether CCM reduces AF burden, PVC burden, sustained VT/VF, ATP, or appropriate ICD shocks; whether any potential effect is independent of HF improvement; and whether benefit differs according to ischemic versus non-ischemic substrate, scar burden, LVEF, AF status, or baseline arrhythmia phenotype. The current evidence base and the corresponding research priorities are summarized in Table 2.

**Figure 1:**
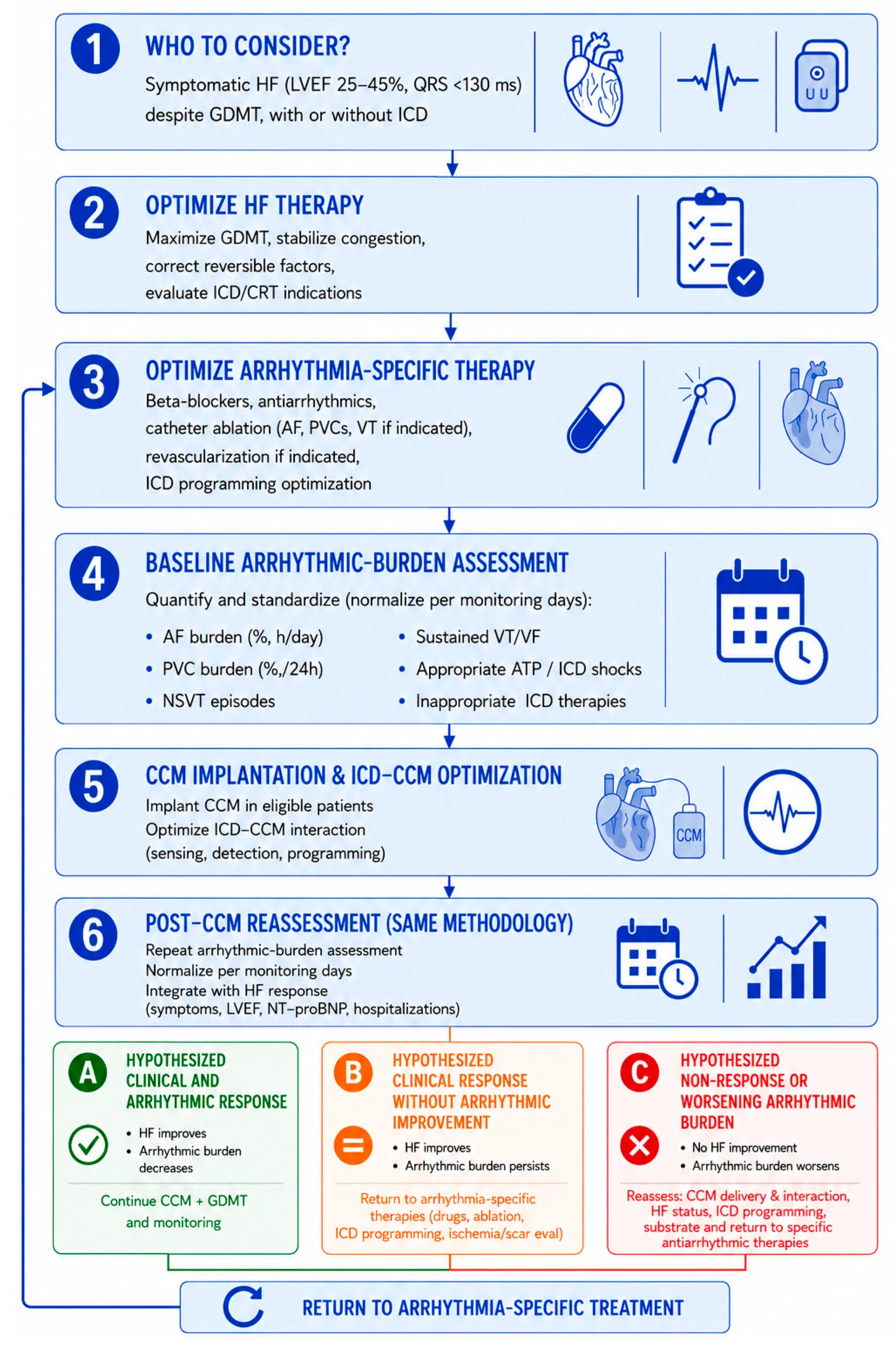
Proposed hypothesis-generating clinical framework for CCM candidate selection and arrhythmic-burden assessment. Patients with symptomatic HF and arrhythmic vulnerability should first undergo GDMT optimization, clinical stabilization, and evaluation of ICD/CRT indications. Arrhythmia-specific treatment should also be optimized before CCM implantation. Arrhythmic burden should be quantified using rhythm-specific metrics, matched pre- and post-CCM monitoring windows, and consistent device programming. Post-implant reassessment should integrate rhythm outcomes with heart failure response. We therefore propose three hypothesized response patterns, which require prospective validation and should not be interpreted as evidence of established antiarrhythmic efficacy. Among these, persistent or worsening arrhythmic burden should prompt reassessment of CCM delivery, ICD–CCM interaction, HF status, ICD programming, arrhythmogenic substrate, and the need for specific antiarrhythmic therapies. Figure created by the authors using BioRender.com. Abbreviations are listed in the Abbreviations section.

This gap is clinically relevant because many CCM candidates have advanced myocardial disease, high arrhythmic vulnerability, and frequent exposure to defibrillator therapies. A reduction in arrhythmic burden could improve symptoms and quality of life, reduce psychological distress and ICD shock burden, and potentially lower hospitalization risk and healthcare resource use. However, without dedicated arrhythmia-focused studies, CCM cannot currently be positioned as an antiarrhythmic or arrhythmia-modifying intervention. Accordingly, the concept of an arrhythmic response to CCM deserves prospective evaluation. Reductions in arrhythmic burden after CCM may not necessarily parallel conventional clinical response and could identify a subgroup with more pronounced modification of the arrhythmogenic substrate. This response may be influenced by scar extent, border-zone architecture, fibrosis distribution, conduction slowing, baseline arrhythmic phenotype, CCM delivery, and the degree of reverse remodeling. Future studies should therefore distinguish clinical response from arrhythmic response and determine whether an integrated clinical–arrhythmic-responder phenotype provides incremental prognostic information. Next-generation studies should move beyond safety reporting and be designed around prespecified arrhythmic endpoints. Arrhythmic burden should be quantified separately by rhythm phenotype: AF burden as the percentage of monitored time in AF, PVC burden as the percentage of total beats, and NSVT, VT/VF, ICD therapies as adjudicated event rates normalized to monitoring or device-observation time. Pre- and post-CCM assessments should use matched observation windows and consistent device programming (Figure 1). Arrhythmic data should also be interpreted alongside markers of HF trajectory and myocardial substrate, including serial echocardiographic remodeling, CMR-defined scar or fibrosis, natriuretic peptides, GDMT dose, antiarrhythmic exposure, renal function, electrolyte profile, congestion status, and HF events.

Moreover, future translational and prospective observational studies should clarify the molecular, electrophysiological, and substrate-level changes associated with CCM, including ion-channel remodeling, connexin-43 and gap-junction regulation, and scar-related conduction properties. Human studies should integrate device-detected arrhythmic burden with markers of electrical and anatomical substrate remodeling. These may include QRS-based metrics, electroanatomical or high-density mapping, non-invasive electrocardiographic mapping, and CMR-based tissue characterization to evaluate changes in scar burden, border-zone architecture, fibrosis distribution, and conduction heterogeneity after CCM therapy [52,53]. Such an integrated framework is essential to distinguish direct antiarrhythmic effects from substrate-mediated arrhythmia modulation related to reverse electromechanical remodeling and HF stabilization.

**Table 2:** Evidence map and future perspectives for CCM and arrhythmic burden.

Clinical Question	Current Evidence	Future Perspective
Proarrhythmic?	No consistent increase in ventricular or supraventricular tachyarrhythmias in early Holter/device-safety data; CCM appears electrically safe when correctly delivered.	Continue systematic safety monitoring in contemporary cohorts, including ICD/S-ICD, AF, higher-LVEF, and integrated CCM-ICD populations.
How should arrhythmic burden be defined?	No standardized definition of arrhythmic burden has been used in CCM studies, and endpoints vary across Holter, ICD, loop recorder, and clinical-event reporting.	Define harmonized endpoints for AF burden, PVC burden, sustained VT/VF, ATP, appropriate and inappropriate shocks, and composite ventricular arrhythmic burden, all normalized to monitoring or device-observation time.
AF burden reduced?	Feasibility in AF is supported, especially with atrial-lead-independent systems, but AF-burden reduction is limited to isolated case-based observations.	Use continuous or device-detected AF monitoring before and after CCM, with standardized assessment of AF burden, recurrence, rhythm-control therapy, and HF status.
PVC/NSVT burden reduced?	No prospective study has systematically quantified PVC or NSVT burden before and after CCM; reduction is biologically plausible but unproven.	Use Holter, patch, ICD, or integrated-device monitoring with standardized baseline and post-activation windows, normalized to monitoring time.
Sustained VT/VF reduced?	Preclinical models suggest reduced VT inducibility and improved electrical remodeling; clinical evidence is limited to case reports and indirect observations.	Prospective ICD-based studies should assess sustained VT/VF timing after activation and independence from HF improvement or medication changes.
ATP/appropriate shocks reduced?	Not proven. Observational studies report ICD therapies during follow-up; one case report suggests reduction, but no study tested ICD therapies as an efficacy endpoint.	Harmonize ICD programming, adjudicate ATP/shocks independently, and normalize event rates to device-observation time.
Inappropriate therapies reduced?	Crosstalk testing and programming may prevent oversensing, misclassification, and inappropriate therapies; this is device optimization, not arrhythmia suppression.	Evaluate inappropriate shocks, oversensing, sensing-vector changes, stored electrograms, and CCM-ICD/S-ICD programming effects on therapy burden.
Safe ICD/S-ICD coexistence?	Combined CCM-ICD/S-ICD experience supports feasibility when crosstalk, sensing, defibrillation testing, stored electrograms, and programming are managed.	Define standardized protocols for interaction testing, device programming, and long-term surveillance.
Integrated CCM-ICD platforms?	Investigational systems may unify CCM delivery, VT/VF detection, ATP/shock therapy, and rhythm monitoring.	Leverage integrated platforms for standardized sensing, synchronized delivery, continuous burden assessment, and adjudicated VT/VF, ATP, and shocks.
Independent of HF improvement?	No direct evidence separates direct electrophysiological effects from substrate-mediated effects related to reverse remodeling and HF stabilization.	Integrate rhythm burden with serial LVEF/LV volumes, CMR scar/fibrosis, natriuretic peptides, congestion, GDMT, antiarrhythmics, ablation, revascularization, and HF events.
Direct modifications of the conduction substrate and/or gap-junction remodeling?	Preclinical data suggest that CCM may restore potassium-channel and gap-junction proteins, including connexin-43, and reduce VT inducibility. However, human evidence linking CCM to improved cell-to-cell coupling, conduction velocity, conduction heterogeneity, or scar-related conduction properties remains lacking.	Future translational and prospective observational studies should integrate device-detected arrhythmic burden with CMR-based scar characterization, electroanatomical or high-density mapping, non-invasive electrocardiographic mapping to assess conduction properties, and, where feasible, molecular assessment of ion-channel and gap-junction remodeling.
Which patients may derive arrhythmic benefit?	No study has identified clinical, electrical, imaging, or arrhythmic phenotypes associated with greater arrhythmic-burden reduction after CCM.	Evaluate predictors of arrhythmic response, including ischemic versus non-ischemic etiology, scar burden, LVEF, LV volumes, QRS duration, NT-proBNP, AF status, PVC/NSVT burden, ICD therapies, autonomic markers, and degree of reverse remodeling.
Does arrhythmic-burden reduction translate into clinical benefit?	No study has tested whether arrhythmic-burden reduction after CCM is associated with fewer HF hospitalizations, fewer ICD shocks, improved quality of life, or better survival.	Link rhythm endpoints to patient-centered and prognostic outcomes, including symptoms, quality of life, exercise capacity, HF hospitalization, ICD shock burden, mortality, and healthcare resource use.
Who is the CCM arrhythmic responder?	No study has prospectively defined an arrhythmic-responder phenotype after CCM. Available evidence does not clarify whether arrhythmic-burden reduction parallels global HF improvement or reflects deeper modification of the arrhythmogenic substrate.	Future studies should distinguish clinical responders from arrhythmic responders and evaluate predictors of arrhythmic response, including scar extent, border-zone architecture, fibrosis distribution, conduction slowing, baseline arrhythmic phenotype, CCM delivery, reverse remodeling, and device-detected arrhythmic burden.

Abbreviations are listed in the Abbreviations section.

## 6. Conclusions

CCM has a biologically plausible but unproven role in arrhythmia modulation. Current evidence supports CCM as an electrically safe, non-proarrhythmic therapy that improves symptoms, functional capacity, and health-related quality of life in selected patients with HF. Direct evidence for reduced atrial or ventricular arrhythmic burden remains very limited and largely case-based.

Any antiarrhythmic signal observed after CCM may reflect direct electrophysiological modulation, indirect improvement in the HF substrate, or non-causal factors such as GDMT optimization, antiarrhythmic drug changes, ablation, revascularization, electrolyte correction, or device reprogramming. Based on current evidence, substrate improvement appears the most plausible explanation, but whether CCM reduces arrhythmic burden independently of remodeling, hemodynamics, and clinical status remains unknown.

Until dedicated prospective studies with standardized rhythm monitoring and adjudicated device endpoints are available, CCM should be regarded as an electrically safe and biologically plausible substrate-modifying therapy, not as an established antiarrhythmic intervention.

## Data Availability

No new data were created or analyzed in this study. Data sharing is not applicable to this article.

## Abbreviations

The following abbreviations are used in this manuscript:

6MWD	6 min walk distance
AF	Atrial fibrillation
ATP	Antitachycardia pacing
cAMP	Cyclic adenosine monophosphate
CCM	Cardiac contractility modulation
CMR	Cardiac magnetic resonance
CRT	Cardiac resynchronization therapy
ECG	Electrocardiogram
GDMT	Guideline-directed medical therapy
GLS	Global longitudinal strain
HF	Heart failure
HFpEF	Heart failure with preserved ejection fraction
HFmrEF	Heart failure with mildly reduced ejection fraction
HFrEF	Heart failure with reduced ejection fraction
ICD	Implantable cardioverter-defibrillator
LV	Left ventricular/left ventricle
LVEF	Left ventricular ejection fraction
MAGGIC	Meta-Analysis Global Group in Chronic Heart Failure
MLWHFQ	Minnesota Living with Heart Failure Questionnaire
NCX	Sodium–calcium exchanger
NSVT	Non-sustained ventricular tachycardia
NT-proBNP	N-terminal pro-B-type natriuretic peptide
NYHA	New York Heart Association
PLN	Phospholamban
PVC	Premature ventricular complex
QoL	Quality of life
RCT	Randomized controlled trial
RyR2	Ryanodine receptor-2
S-ICD	Subcutaneous implantable cardioverter-defibrillator
SERCA2a	Sarcoplasmic/endoplasmic reticulum calcium ATPase 2a
TGF-β1	Transforming growth factor beta 1
VAT	Ventilatory anaerobic threshold
VO_2_	Oxygen consumption
VT/VF	Ventricular tachycardia/ventricular fibrillation
